# Lasing properties and carrier dynamics of CsPbBr_3_ perovskite nanocrystal vertical-cavity surface-emitting laser

**DOI:** 10.1515/nanoph-2023-0081

**Published:** 2023-05-03

**Authors:** Yawen He, Zhan Su, Fuyi Cao, Zhenghao Cao, Yuejun Liu, Chunhu Zhao, Guoen Weng, Xiaobo Hu, Jiahua Tao, Junhao Chu, Hidefumi Akiyama, Shaoqiang Chen

**Affiliations:** State Key Laboratory of Precision Spectroscopy, Department of Electronic Engineering, East China Normal University, 500 Dongchuan Road, Shanghai 200241, China; Institute for Solid State Physics, The University of Tokyo, 5-1-5 Kashiwanoha, Kashiwa, Chiba 277-8581, Japan

**Keywords:** carrier dynamics, CsPbBr_3_ nanocrystal, perovskite, vertical-cavity surface-emitting laser

## Abstract

All-inorganic lead halide perovskite nanocrystals (NCs) have been widely investigated as highly promising optical gain materials due to their compelling electrical and optical properties. Although many efforts have been carried out, a deep understanding of perovskite NC vertical-cavity surface-emitting lasers (VCSELs) is elusive, which is very important in the development of photoelectronic integrated circuits. Along these lines, in this work, a low lasing threshold (22 μJ/cm^2^) single-mode VCSEL consisting of CsPbBr_3_ NCs film and two distributed Bragg reflectors was successfully constructed. The CsPbBr_3_ NCs were synthesized by using the supersaturated recrystallization method. Interestingly, benefiting from the strong coupling between the active layer and the optical field in the cavity, a single-mode lasing at 527 nm was demonstrated under femtosecond optical pumping. The carrier dynamics of the perovskite NC VCSEL was also thoroughly investigated by performing pump intensity-dependent time-resolved photoluminescence measurements. The typical gain-switching phenomenon was observed with an ultrafast decay of the laser pulse of ∼10 ps. Our work provides valuable insights for the implementation of the CsPbBr_3_ NC VCSEL for various optoelectronic applications.

## 1 Introduction

All-inorganic lead halide perovskites, as an emerging class of semiconductor materials, have excellent electrical and optical properties, such as high defect tolerance, long carrier lifetime, high carrier mobility, high optical absorption coefficient, and low defect density [1–4]. Moreover, inorganic perovskites possess better stability at high temperature and high humidity conditions, which prolongs the device lifetime compared to those of organic–inorganic hybrid perovskites [5–7]. Therefore, inorganic perovskites are emerging as a promising photonic material in the fields of photoelectric detection, photovoltaic, and illumination, especially as an optical gain material [8–11]. As far as nanocrystals (NCs) are concerned, it has been also demonstrated that they have intrinsically high photoluminescence (PL) quantum yield as an active medium. The underlying origins of this effect are associated with the fact that both the electrons and holes in NCs are strictly confined in a small volume and readily recombine radiatively due to the quantum confinement effect [12, 13]. In addition, the electron and hole energy levels of NCs are quantized [14], resulting in light emission with a narrow full width at half maximum (FWHM) and a broadband tunable wavelength range. By combining the comparative advantages of all-inorganic perovskites and NCs, CsPbBr_3_ NC was proposed as an ideal material for amplifying spontaneous emission and the laser emission.

The previously reported works in the literature studies have mainly focused on the perovskite micro/nano-material itself as the gain medium. Although the fabrication of optical pump lasers has been reported in perovskite nanowires and microdisks [15–20], the fabricated lasers are mostly multi-mode lasers. The mode competition between the different modes affects not only the stability of the output energy, but also the population inversion in the laser cavity, which inevitably increases the energy consumption of the laser. Besides, the multi-mode laser reduces the coherence and the monochromaticity properties of the laser, which is unfavorable for the utilization of lasers in photoelectronic integrated circuits and photonic integrated circuits. One of the most effective ways to reduce the longitudinal modes is the implementation of a submicron optical cavity and a coupled cavity structure [21, 22]. Thus, the design of the external resonant cavity is very important for the operation of single-mode lasers, which can significantly improve the compatibility of the lasers in integrated optoelectronic systems.

Vertical-cavity surface-emitting lasers (VCSELs) employ two highly reflective and parallel distributed Bragg reflectors (DBRs) as the cavity, with a gain medium sandwiched between them for stimulated emission. VCSELs exhibit low threshold current, high quantum efficiency, and high-speed modulation at low current values due to the small size of their resonant cavity [23, 24]. In addition, the symmetric transverse structure and surface emission characteristics of VCSELs result in a circular and low-divergence emitting beam, which facilitates the coupling into optical devices. Owing to these properties, VCSELs have been extensively used as a laser source in various applications, such as optical communication, optical storage, laser display, and laser sensing [25–27]. In recent years, the development of single-mode VCSELs with semiconductor materials as the gain medium has been reported in the literatures [28–33]. However, the properties of inorganic perovskite NC VCSELs have been scarcely examined, especially when the optical gain dynamics of VCSELs is concerned. However, the latter is crucial for the wide applications of perovskite lasers in the future.

Under this direction, in this work, high-quality CsPbBr_3_ NCs were synthesized by using the supersaturated recrystallization method [34]. On top of that, a green perovskite VCSEL using CsPbBr_3_ NCs as the gain medium was fabricated. The VCSEL exhibited a single-mode lasing under femtosecond optical pumping, with an emission wavelength at 527 nm, and an FWHM of 0.49 nm, corresponding to a high quality factor of 1076. Due to the strong electron-photon interaction in the cavity, a low lasing threshold of 22 μJ/cm^2^ was also demonstrated. The carrier dynamics of the CsPbBr_3_ NC VCSEL was also systematically investigated using a streak camera system. The gain-switching characteristics and the down-chirp effect of the output pulses were clearly observed and physically explained. Our results provide significant guidance for high-performance single-mode perovskite lasers and a better understanding of the light–matter interaction and carrier dynamics in the perovskite microcavity.

## 2 Results and discussion

**Figure 1:**
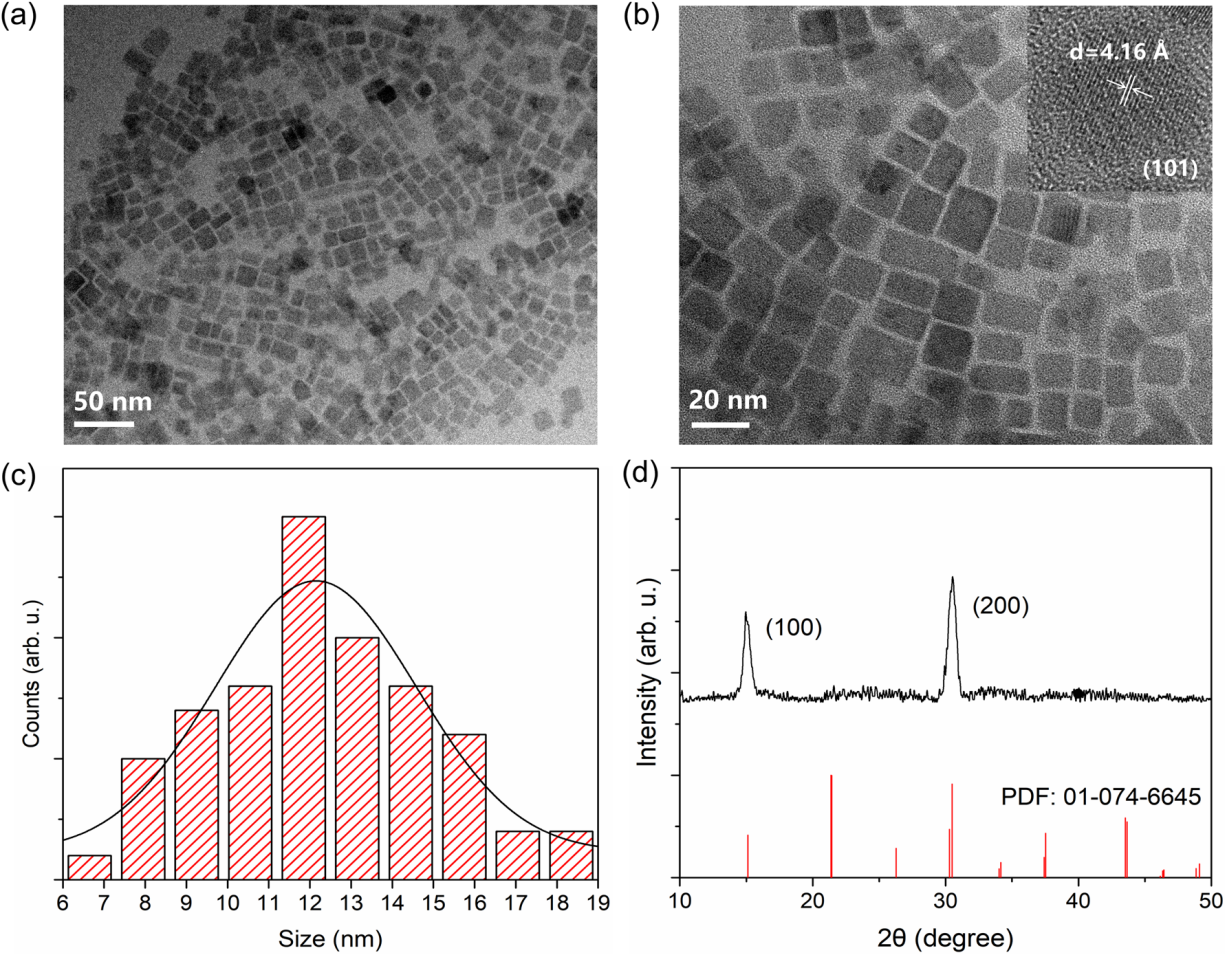
Morphology and crystal structure of CsPbBr_3_ NCs. (a, b) TEM images and HRTEM image (the inset in Figure 1(b)) of the CsPbBr_3_ NCs. (c) The size distribution of the CsPbBr_3_ NCs calculated from Figure 1(b). (d) XRD pattern of the CsPbBr_3_ NCs film. The standard XRD pattern of tetragonal CsPbBr_3_ (PDF No. 01-074-6645) is also presented for comparison.

The CsPbBr_3_ NC solution was synthesized by using the supersaturated recrystallization method. To characterize the morphology and crystal phase of the synthesized CsPbBr_3_ NCs, transmission electron microscopy (TEM) measurements were performed, as shown in Figure 1(a and b). From the acquired results, it is revealed that the CsPbBr_3_ NCs have cuboidal morphologies. By analysing the randomly selected region of the TEM image, the average particle size of the CsPbBr_3_ NCs is measured to be 12 nm, which is consistent with that of a typical nanocrystal. As can be also observed from Figure 1(c), the size distribution of NCs is relatively wide. However, more than 60 % of the NCs is between 10 and 15 nm.

From the high-resolution TEM (HRTEM, see the inset in Figure 1(b)) image, the crystal structure of a single CsPbBr_3_ NC particle can be examined. More specifically, clear stripes can be noticed, indicating that the sample has good crystallinity. The crystal phase of NCs was also analysed by HRTEM. The interplanar distance of the selected NC is 0.416 nm, corresponding to the (101) crystal plane of tetragonal CsPbBr_3_. X-ray diffraction (XRD) spectrum was also collected to further investigate the crystal structure of the CsPbBr_3_ NCs, as illustrated in Figure 1(d). As can be seen, the CsPbBr_3_ NCs have diffraction peaks at 2*θ* of 15.09° and 30.55°, corresponding to the (100) and (200) crystal planes of the tetragonal phase (PDF No. 01-074-6645). Therefore, based on the results of the HRTEM and XRD measurements, the synthesized CsPbBr_3_ NCs are found to adopt a tetragonal phase of the space group *P*4*mm*. It is well known that the CsPbBr_3_ NCs have orthorhombic, tetragonal, and cubic phases, among which the cubic phase is crystallized at high temperature values [35, 36]. The recrystallization temperature of our method was 60 °C, which was much lower than the synthesis temperature in the thermal injection method [37]. Therefore, the low synthesis temperature may be responsible for the tetragonal rather than the cubic crystal structure of the prepared CsPbBr_3_ NCs. In addition, it can be noticed that the FWHM of the two diffraction peaks is relatively large [11], which is consistent with the nanoscale grain size according to the Scherrer equation.

**Figure 2:**
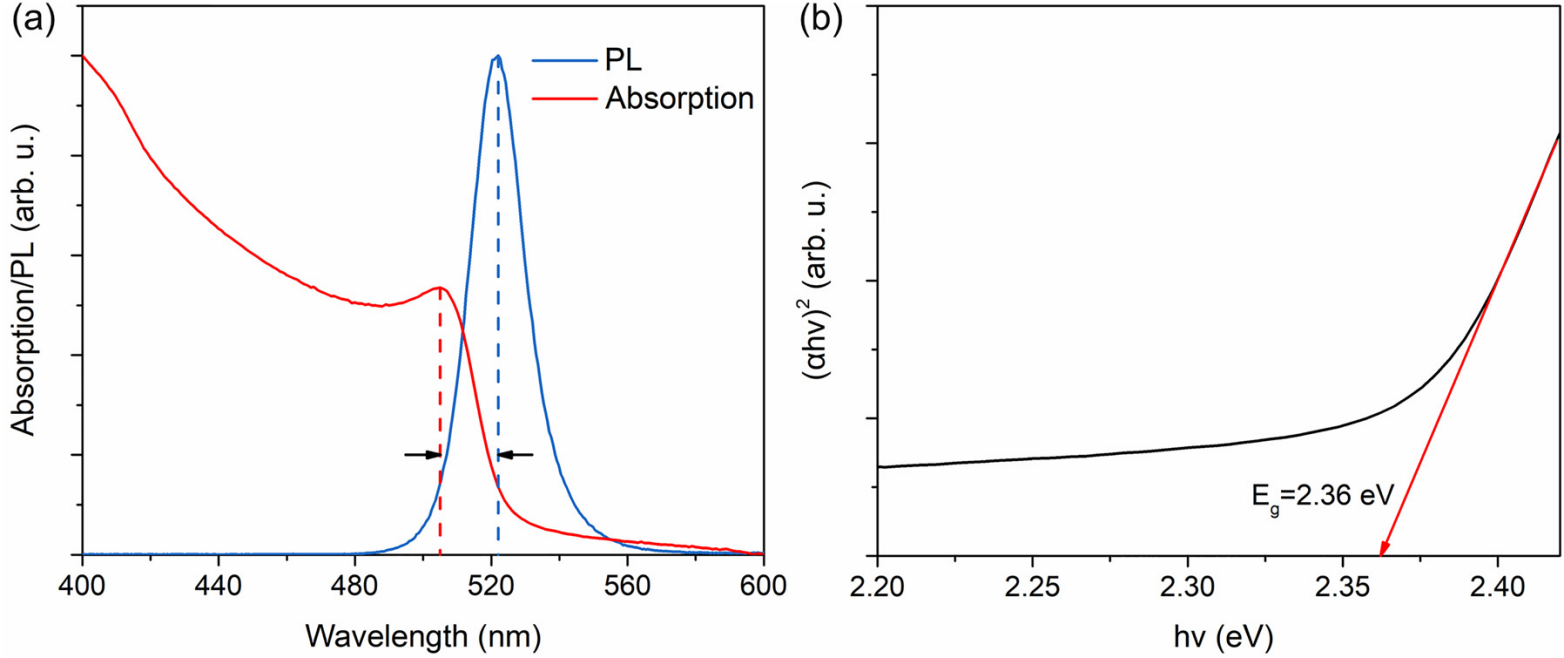
Optical properties of CsPbBr_3_ NCs. (a) PL spectrum, UV–vis absorption spectrum of the CsPbBr_3_ NCs film and their relative positions in wavelength. (b) Tauc plot of the UV–vis absorption spectrum (Figure 2(a)) and calculation of the band gap energy. The band gap energy refers to the intersection of the red line and the *x*-axis, which is 2.36 eV.

Figure 2 depicts the PL and ultraviolet–visible (UV–vis) absorption spectra of the synthesized CsPbBr_3_ NCs. When pumped with 400 nm laser pulses, the CsPbBr_3_ NCs film exhibits a green light emission visible to the naked eye. The PL spectrum (Figure 2(a)) displays an emission peak at 522 nm with a relatively wide FWHM of 20.5 nm, demonstrating the typical spontaneous emission process. Additionally, the PL peak exhibits a Stokes shift of 17 nm from the absorption edge located at 505 nm, which can be attributed to a size-dependent quantum confinement effect [38]. To calculate the band gap of the CsPbBr_3_ NCs, the Tauc plot method [39] is applied to the absorption spectrum, as can be observed in Figure 2(b). The band gap energy is accordingly determined to be 2.36 eV, which is only slightly smaller than the energy corresponding to the PL peak, suggesting that the spontaneous emission of the CsPbBr_3_ NCs film is caused by the radiative recombination of carriers between the conduction band edge and the valence band edge.

**Figure 3:**
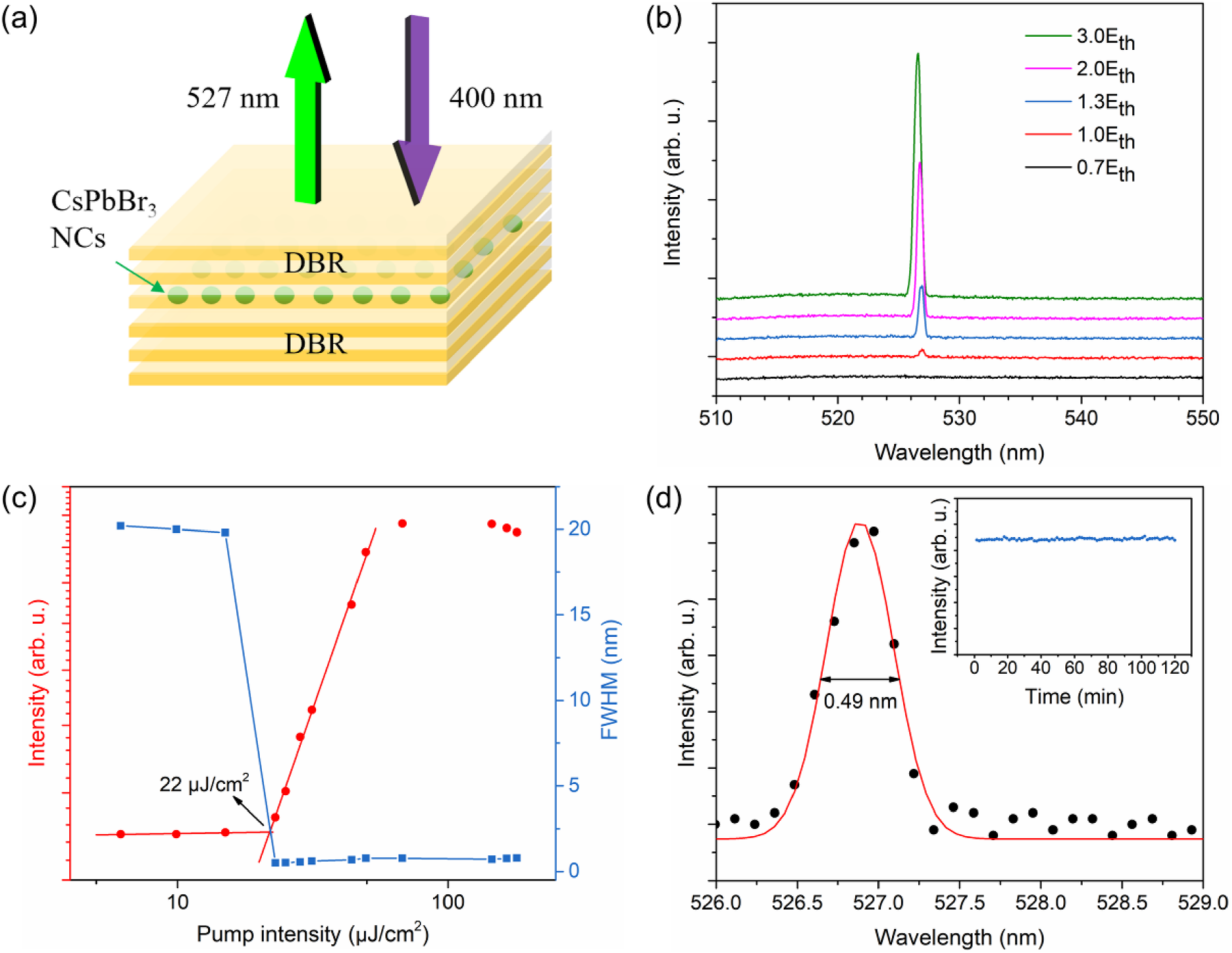
Diagram and lasing properties of CsPbBr_3_ NC VCSEL. (a) Schematic diagram of the CsPbBr_3_ NC VCSEL. The purple arrow refers to the pump source (400 nm). The green arrow refers to the output lasing (527 nm). (b) Evolution of the emission spectra with increasing pump intensity of the CsPbBr_3_ NC VCSEL. (c) L-L curve for the CsPbBr_3_ NC VCSEL, showing a lasing threshold at 22 μJ/cm^2^, which is defined by the intersection of two red lines. The blue squares correspond to the variation of the FWHM of the emission spectra with pump intensity. (d) The lasing spectrum (black dots) at a pump intensity of 1.1*E*_th_. The spectrum can be fitted with a Gaussian curve (red curve). The inset shows the stability characterization of the CsPbBr_3_ NC VCSEL under 1.1*E*_th_.

Figure 3(a) depicts the schematic diagram of the VCSEL using the CsPbBr_3_ NCs applied as the gain medium for laser emission. The resonant cavity is composed of CsPbBr_3_ NCs film sandwiched between two custom DBR mirrors. Figure 3(b) illustrates the PL spectra under different pump intensities. A narrow peak at ∼527 nm appears as the pump intensity increases, indicating the evolvement from spontaneous emission to stimulated emission. To calculate the threshold pump intensity, the relationship between the output lasing intensity and the pump intensity is extracted in Figure 3(c). It is illustrated that the data can be fitted with two straight lines, indicating a rapid increase in the emission at the threshold energy density. The threshold pump intensity is finally identified to be 22 μJ/cm^2^. Due to the enhanced quantum confinement in the CsPbBr_3_ NCs, the nonradiative recombination can result from the strong Auger recombination and exciton–phonon interaction [40], thus increasing the lasing threshold. The Auger recombination rate is proportional to the exciton binding energy of a material, which can be reduced by introducing high-polar organic cations or inorganic ligands into CsPbBr_3_ NCs [41, 42]. In addition, large-size vacancy clusters can be an effective approach to tune the exciton–phonon coupling in low-dimensional semiconductors [43], further lowering the threshold.

A sudden decrease in FWHM is also observed under the threshold pump intensity (see Figure 3(c)), further verifying the achievement of lasing. The lasing spectrum can be well fitted with a Gaussian function with an emission wavelength at 527 nm and an FWHM of 0.49 nm, as depicted in Figure 3(d). The laser emission peak shows a red shift of 5 nm compared to the spontaneous emission peak at 522 nm. The red shift may be induced by the self-absorption in single-exciton lasing or bi-exciton recombination [44]. The quality factor *Q* of the laser is calculated to be *Q* = *λ*/*δλ* = 1076 (where *λ* and *δλ* refer to the emission wavelength and the FWHM, respectively), which is larger than the value of the hybrid perovskite microstructure reported previously [45, 46].

To investigate the long-term reliability of the device, the stability test was performed when the CsPbBr_3_ NC VCSEL was operated under the pump intensity of 1.1*E*_th_ (*E*_th_ is the threshold pump intensity). As shown in the inset of Figure 3(d), the lasing intensity remains at the same level within 2 h of operation, indicating good photostability and thermal stability of the VCSEL.

**Figure 4:**
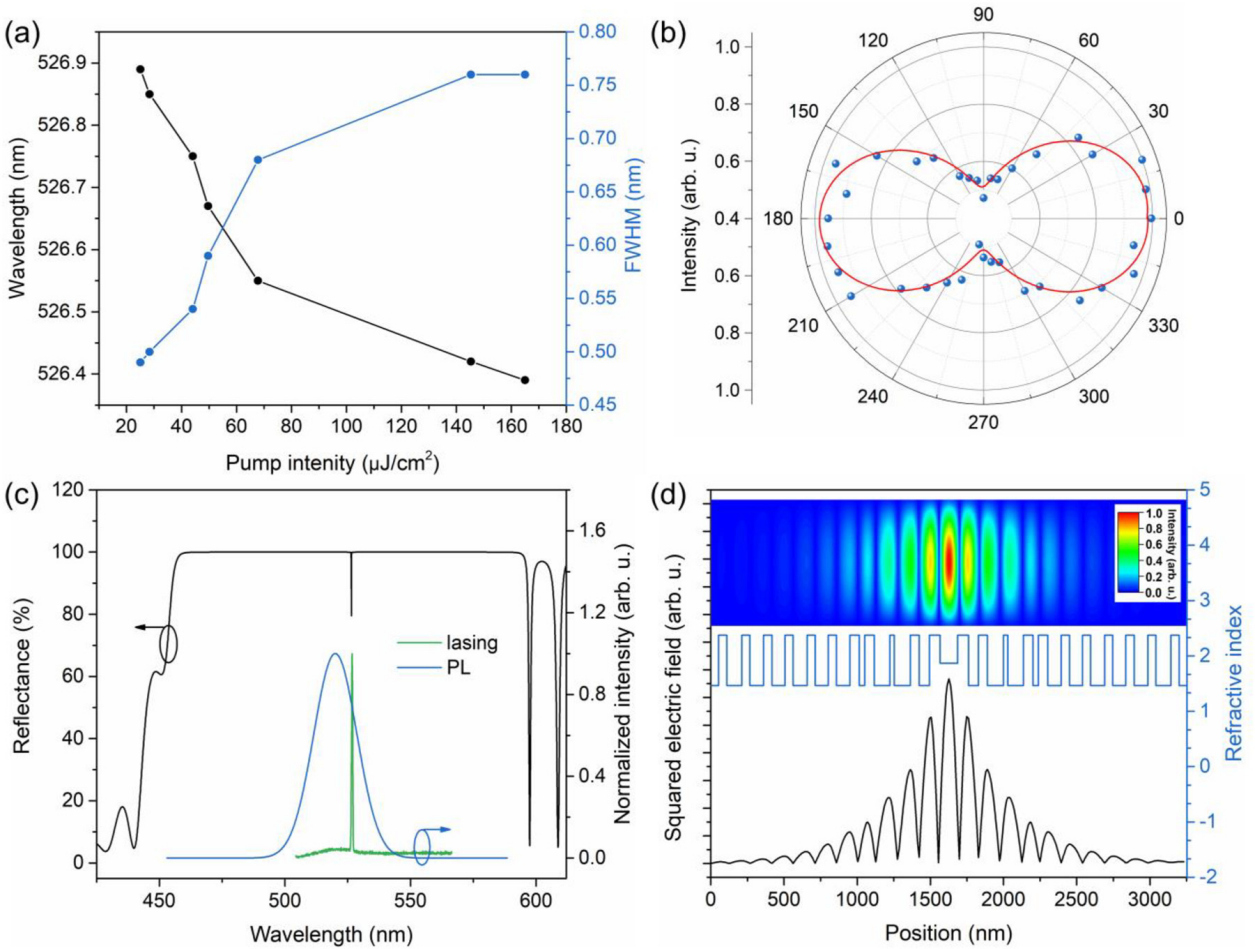
Laser characteristics and optical field distribution of CsPbBr_3_ NC VCSEL. (a) Pump intensity-dependent lasing peak wavelength and FWHM. (b) Normalized lasing peak intensity versus polarization angle. Blue dots refer to experimental data, while the red line refers to fitted data. (c) The measured lasing spectrum, PL spectrum and the calculated reflection spectroscopy of the CsPbBr_3_ NC VCSEL. (d) The stimulated cross-sectional squared electric field distribution versus refractive index of the CsPbBr_3_ NC VCSEL. The top inset represents the corresponding stimulated optical field distribution.

Figure 4(a) shows the variation of lasing peak and FWHM as a function of the pump intensity. It can be seen that the lasing peak exhibits a slight blue shift of 0.5 nm and the FWHM becomes larger with elevating pump intensity above the lasing threshold. The blue shift of the peak wavelength can be explained by taking into account the carrier dynamics. Particularly, with the increase in the carrier density, the refractive index (*n*) of the CsPbBr_3_ NCs decreases, leading to the blue shift of the peak wavelength (*λ*_mode_) according to the mode condition *λ*_mode_/*n* = const [47]. In addition, the expansion of the FWHM can be attributed to the intensification of the lattice vibration based on the photo-induced thermal effect and the accumulation of carriers [48]. As was reported above, a single-mode lasing CsPbBr_3_ NC VCSEL was achieved.

The relationship between the normalized output lasing peak intensity and the polarization angle is almost consistent with the cos^2^ *θ* function, as displayed in Figure 4(b), implying a polarization excitation response of the device. The degree of polarization (DOP) of our device is calculated to be 36 % according to the relationship DOP = (*I*_max_ − *I*_min_)/(*I*_max_ + *I*_min_) × 100 %. The polarization property can be attributed to the asymmetric structure of the CsPbBr_3_ NCs structure. The distorted cubic structure corresponding to the tetragonal phase would break the space inversion symmetry, leading to a preferred direction of the lowest optical transition dipole moment [49, 50], which can be a possible explanation for the polarized emission. In addition to the intrinsic polarized emission of the NCs, the polarized emission of the VCSEL is also modulated by the cavity [51].

The reflection spectroscopy of the VCSEL with a cavity mode at 527 nm is also simulated (Figure 4(c)), from which the cavity length is calculated to be 120 nm. The high reflection band covers the broad PL spectral range to achieve resonate stimulated emission with high modal gain. It is worth noting that the wavelength and the FWHM of the PL peak can be changed due to the different size and quality of the NCs at different excitation positions. However, the lasing wavelength of the VCSEL is almost determined by the cavity length alone, independent of the properties of the NCs. The cavity mode of the VCSEL can be controlled by adjusting the cavity length [52]. In addition, the modal gain in the VCSEL is greatly increased when the cavity mode overlaps with the PL peak, further lowering the lasing threshold.

Moreover, the optical field (squared electric field) distribution of the lasing mode in the cavity is explored by using the transfer matrix method (TMM) [51], together with the refractive index profile of the VCSEL structure, as shown in Figure 4(d). The inset illustrates the corresponding contour map of the optical field distribution. As can be clearly seen, the CsPbBr_3_ NC active layer is located at the antinode of the optical wave, which contributes to the strong coupling between the NC layer (electrons) and the optical field (photons), namely the strong electron–photon interactions in the cavity. As a consequence, the modal gain in the VCSEL is greatly enhanced [53], resulting in low threshold single-mode lasing. Note that the standing-wave optical field penetrates into the DBR region and rapidly drops off (see Figure 4(d)). As a result, the effective cavity length should be longer than the thickness of the CsPbBr_3_ NC active layer.

**Figure 5:**
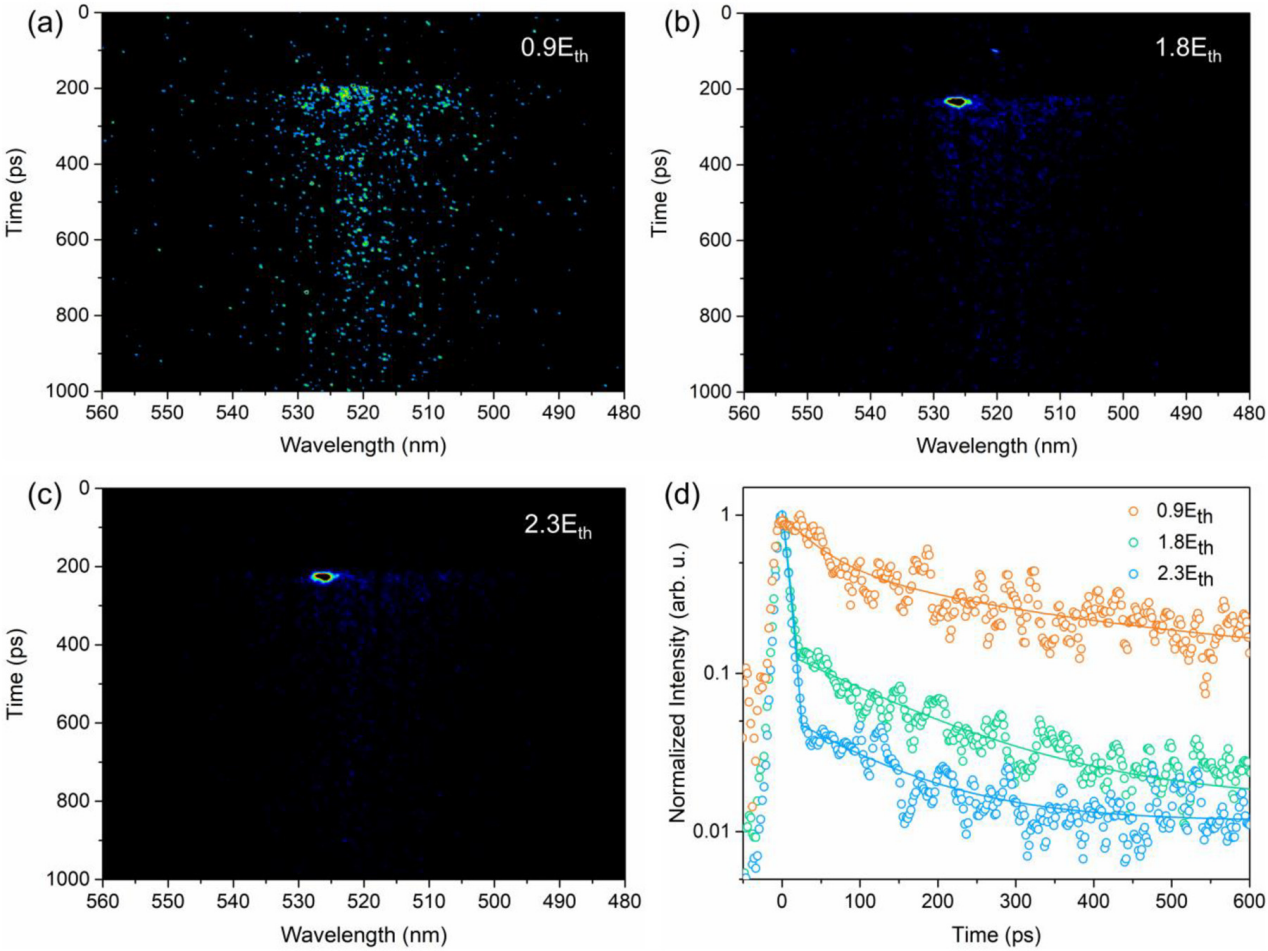
Carrier dynamics of CsPbBr_3_ NC VCSEL. (a–c) Streak camera images of the CsPbBr_3_ NC VCSEL under various pump intensities. The corresponding pump intensities are displayed at the top-right of the graphs. (d) Pump intensity-dependent TRPL decay curves of the VCSEL. The hollow dots correspond to the experimental data. The curves correspond to the fitting results.

To gain a deeper insight into the dynamics of photogenerated carriers during lasing emission, time-resolved PL (TRPL) spectroscopy measurements were performed. Figure 5(a–c) depict the streak camera images under pump intensities of 0.9*E*_th_, 1.8*E*_th_, and 2.3*E*_th_, respectively. As can be seen from Figure 5(a), the CsPbBr_3_ NC VCSEL exhibits a spontaneous emission over a wide wavelength range below the threshold pump intensity (0.9*E*_th_). According to Figure 5(b–c), when the pump intensity is above the lasing threshold, a narrow emission peak is measured at a wavelength of ∼527 nm. Meanwhile, the spontaneous emission becomes relatively weaker, representing that the stimulated emission dominates the emission process. In addition, the lifetime of the laser emission is much shorter than that of the spontaneous emission. The lasing lasts only for a very short time scale (0–40 ps) since its appearance.

The decay dynamics of the excited carriers are manifested in the TRPL decay curve in Figure 5(d). Under 0.9*E*_th_, the decay trace is well-fitted with a double exponential function. The fitting result reveals a fast decay time of 48 ps and a slow decay time of 318 ps. Based on the previously reported work in the literature, the rapid and slow components can be attributed to the surface state and bulk recombination [15], respectively. Above the threshold, the decay trace is split into two single exponential fits, where a fast decay process of stimulated emission and a slow decay process of spontaneous emission can be clearly exhibited. The ultrashort lifetime of 11 ps is observed under 1.8*E*_th_, further confirming the existence of laser emission. The short lifetime of the lasing is likely due to electron-hole recombination and other higher-order processes [54]. It is interesting to notice that the photon lifetime becomes even shorter when the pump intensity reaches 2.3*E*_th_. The ultrafast decay time of about 10 ps implies a high modulation bandwidth (∼GHz) of the VCSEL.

**Figure 6:**
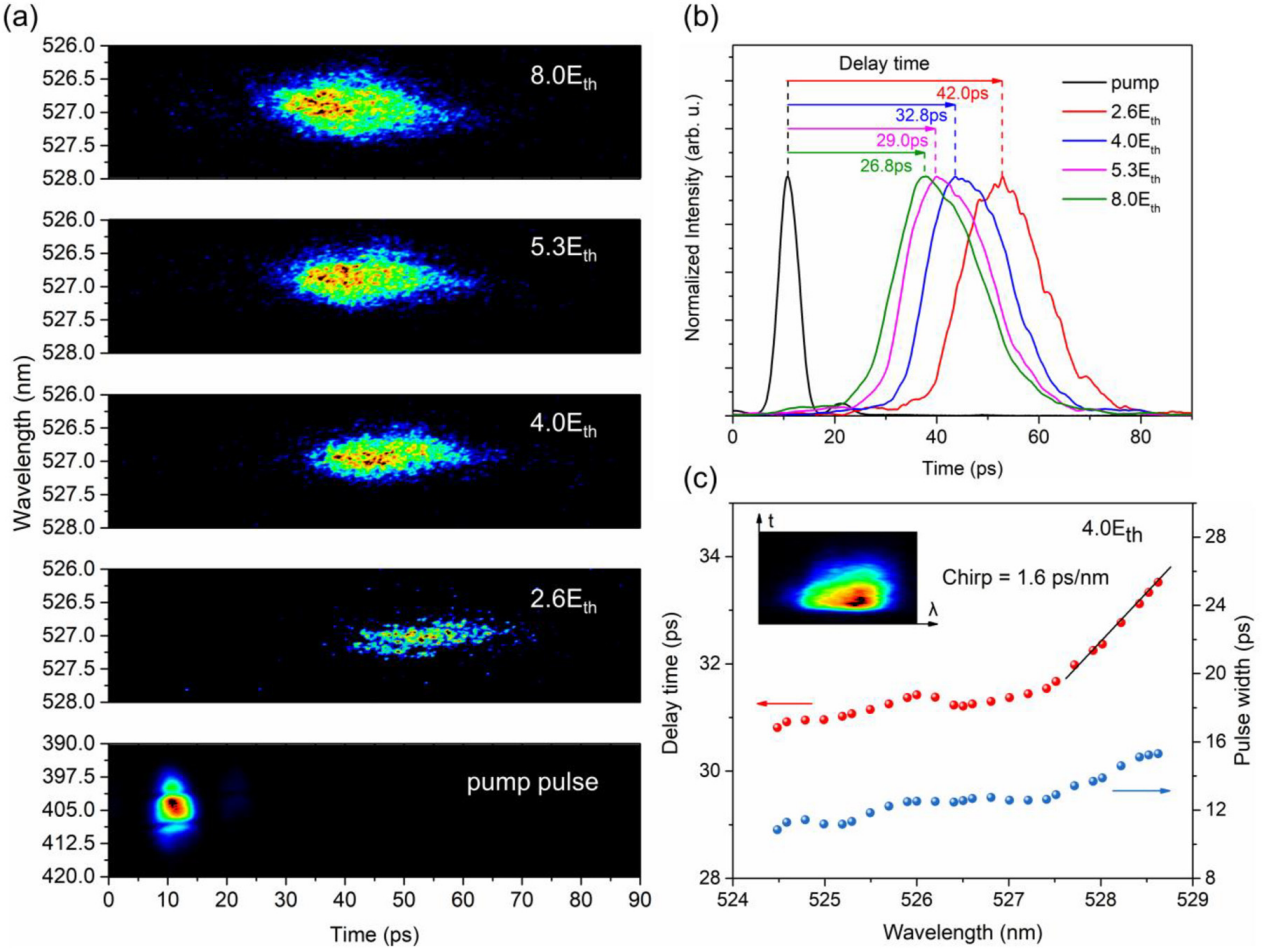
Gain-switching characteristics of CsPbBr_3_ NC VCSEL. (a) Temporally- and spectrally-resolved streak camera images of the VCSEL for various pump intensities above the lasing threshold and the 400 nm pump pulse. The measured pump pulse width is affected by the resolution limit of the streak camera. Therefore, the figure does not represent the actual pump pulse width. (b) Spectrally integrated output lasing pulse waveforms and delay time under different pump intensities, which is extracted from Figure 6(a). (c) Delay time and pulse width of the laser outputs under different wavelengths with a pump intensity of 4.0*E*_th_, which is extracted from the streak camera image (the inset).

In order to further characterize the gain-switching dynamics of the CsPbBr_3_ NC VCSEL, TRPL measurements were further performed under different pump intensities, as shown in Figure 6(a). As can be seen, a laser emission from the VCSEL appears tens of picoseconds after the arrival of the 400 nm pump pulse. It is also obtained from the streak camera image that the laser emission has a blue shift of wavelength and an increasing FWHM as the pump intensity increases, which is consistent with Figure 4(a). The blue shift may be related to the excitons transitioning to electron-hole plasmas (EHPs) via the Mott transition [55]. When the carrier density surpasses the Mott density, the excitons crossover to EHPs, while the laser emission occurs due to the reduction of the Coulomb interaction [56–58]. The carrier density under the threshold pump intensity is 1.58 × 10^18^ cm^−3^, which is calculated by *n*_*p*_ ≈ (*η*·*P*_exc_)/(ℏ*ω*_exc_·*V*_*c*_), where *η* is the PL quantum yield, *P*_exc_ stands for the average pump intensity, ℏ represents the reduced Planck constant, *ω*_exc_ denotes the angular frequency of the pump photon, and *V*_*c*_ refers to the effective volume of the active region. The carrier density is higher than the reported Mott density of CsPbBr_3_ (1.8 × 10^17^–4.7 × 10^17^ cm^−3^) [15], verifying the formation of EHPs in the laser emission. Based on the above-mentioned analysis, it can be concluded that the EHP mechanism may be responsible for the stimulated emission of the CsPbBr_3_ NC VCSEL.

Figure 6(b) plots the spectrally integrated lasing pulse waveforms extracted from Figure 6(a). The delay time refers to the time interval between the output lasing pulse and the pump pulse. As the pump intensity increases from 2.6*E*_th_ to 8.0*E*_th_, the delay time is reduced from 42.0 ps to 26.8 ps, indicating a typical gain-switching phenomenon [59]. With high pump intensity, the input energy is high enough to cause carrier inversion in the gain medium. This non-equilibrium distribution will relax by exciton–phonon interaction [60]. The energy redistribution-induced hot electrons and holes will return to the equilibrium state through the interaction with the CsPbBr_3_ NC lattice. That is to say, the photo-generated carriers return to the bottom of the energy band through the interaction with phonons, where the electron–hole pairs recombine to achieve the lasing operation. As a result, the output lasing pulse has a delay of several picoseconds with respect to the pump pulse. The delay time strongly depends on the pump intensity, since the delay of stimulated emission becomes shorter as the carrier density increases. In addition, all the output pulse widths are shorter than 19.4 ps, similar to the value observed in the CsPbBr_3_ microwire [61]. The short pulse width can be attributed to the carrier heating, which reduces the saturated gain during gain-switching [61–64]. However, due to the short cavity length of the VCSEL, a shorter pulse width is expected in the VCSEL than in the nanowire [25]. The deviation between the experimental results and the expectations may be due to the degradation of the NC quality during the fabrication of the VCSEL. The intrinsic optical quality of the fragile CsPbBr_3_ NCs can be preserved by spin-coating a PMMA layer between the CsPbBr_3_ NCs and the DBR [65]. Nevertheless, it can be concluded that the CsPbBr_3_ NC VCSEL has the potential as an ultrafast laser source. In addition, a shorter pulse width can be achieved by increasing material gain or reducing the cavity length [66, 67].

Figure 6(c) exhibits the delay time and pulse width of the laser outputs under different wavelengths with a pump intensity of 4.0*E*_th_. The delay time and pulse width under each wavelength are extracted from the streak camera image (see the inset). The delay time lengthens by increasing the wavelength (down chirp) and shows an almost linear increase with a slope of 1.6 ps/nm over the wavelength range from 527.4 nm to 528.6 nm. In addition, the pulse width reveals the same upward trend with increasing wavelength, maintaining at ∼12 ps and increasing to ∼15 ps above 527.4 nm. This increase due to the chirp effect makes it difficult to achieve a short pulse width in gain-switching lasers. The generation of chirp is also closely related to the refractive index of the active medium. Therefore, the chirp is inevitable in gain-switching lasers. Still, short pulses can be obtained through chirp elimination techniques, such as spectrum filtering, pulse compression, and pulse shaping [68–71]. The spectral filtering is an important approach to extract ultrashort pulses beyond the photon lifetime limit. As a result, fs-level pulses have been demonstrated in gain-switching semiconductor lasers after spectra filtering [72].

## 3 Conclusions

In summary, a perovskite VCSEL consisting of two parallel DBRs and a CsPbBr_3_ NCs film sandwiched between was demonstrated. A single-mode lasing operation at 527 nm with threshold pump intensity of 22 μJ/cm^2^ was realized. The cavity quality factor was calculated to be 1076. The simulated optical field distribution indicated a strong coupling between the NC active layer and the optical field in the cavity. By using the streak camera, it was observed from the TRPL measurement that the stimulated emission possessed a fast decay time of ∼10 ps. The gain-switching dynamics of the CsPbBr_3_ NC VCSEL was also examined for the first time. It was observed that the delay time under different pump intensities was all shorter than 42.0 ps and the pulse width was shorter than 19.4 ps without any post-processing. Our work paves the way for a better understanding of the gain-switching mechanism of CsPbBr_3_ NC VCSELs, which is very important for VCSEL applications, such as optical storage and ultrafast optical communication.

## 4 Methods

### 4.1 Synthesis of CsPbBr_3_ NCs

Lead bromide (PbBr_2_, 99.999 %), cesium bromide (CsBr, 99.999 %), N,N-dimethylformamide (DMF, AR, 99.5 %), oleic acid (OA, AR), oleylamine (OAm, 80–90 %), and toluene (≥99.5 %) were purchased from Aladdin. The CsPbBr_3_ NC solution was prepared by the supersaturated recrystallization method in atmosphere. PbBr_2_ (0.4 mmol), OA (1 mL), OAm (0.1 mL), and CsBr (0.4 mmol) were added to DMF (10 mL) and stirred until dissolved. Then, 0.5 mL of the precursor solution was rapidly added to toluene (5 mL) while vigorously stirring. A strong green emission was observed under ultraviolet (UV) light immediately after the injection. The above steps were all performed at 60 °C. The NCs and other larger crystals in the above solution were separated by centrifugation at 8000 rpm for 20 min. The supernatant was replaced with toluene and centrifugation was repeated at 10,000 rpm for 15 min. After centrifugation, the supernatant was collected as the CsPbBr_3_ NC solution. The CsPbBr_3_ NC solution was dispersed on a clean glass substrate until the toluene evaporated to form the CsPbBr_3_ NCs film.

### 4.2 Fabrication of CsPbBr_3_ NC VCSEL device

Commercially available DBR mirrors with an alternating TiO_2_/SiO_2_ structure were purchased. The DBR mirrors were successively cleaned with acetone, ethanol and deionized water in the ultrasonic cleaning machine. An appropriate amount of NC solution was dropped on the upper surface of the DBR mirror and waited for the toluene to evaporate. Another DBR was placed in reverse on top of the above DBR to form the VCSEL. The VCSEL was sealed by applying optical epoxy resin to the edges of the DBR mirrors. Finally, the VCSEL was pressed and exposed to UV light for 24 h.

### 4.3 Characterization

The XRD pattern of the as-synthesized CsPbBr_3_ NCs film was measured by a D8-Discover X-ray diffraction spectrometer with Cu Kα radiation. TEM and HRTEM measurements were performed with an FEI Tecnai G2 F30 microscope with an accelerating voltage of 300 kV. The UV–vis absorption spectrum was taken by a Perkin Elmer Lambda 950 UV-vis-NIR spectrometer. A femtosecond laser (400 nm, 35 fs, 1 kHz) was used as the pump source for PL and lasing measurements. The 400 nm pulses were generated by frequency doubling the 800 nm fs pulses using a β-barium borate (BBO) crystal, which were emitted from a Ti:sapphire regenerative amplifier system (Verdi G8, Coherent). The PL and lasing spectra were recorded with an Andor Solis SR303 triple-grating spectrometer. TRPL measurements were acquired with a Hamamatsu streak camera system using the same 800 nm fs laser source. All tests were performed at room temperature.
